# Stigma Attitudes Toward HIV/AIDS From 2011 Through 2023 in Japan: Retrospective Study in Japan

**DOI:** 10.2196/69696

**Published:** 2025-05-12

**Authors:** Yi Piao, Nao Taguchi, Keisuke Harada, Kunihiro Hirahara, Yosuke Takaku, John Austin, KuanYeh Lee, Yui Shiozawa, Yunfei Cheng, Yoji Inoue

**Affiliations:** 1 Gilead Sciences KK Tokyo Japan; 2 Japanese Network of People Living with HIV/AIDS Tokyo Japan; 3 Gilead Sciences (United States) Foster City, CA United States; 4 Deloitte Tohmatsu Consulting LLC Tokyo Japan; 5 Accelight Inc Tokyo Japan

**Keywords:** HIV, machine learning, stigma, social media, Twitter, X

## Abstract

**Background:**

Stigma associated with HIV/AIDS continues to be a major barrier to prevention, management, and care. HIV stigma can negatively influence health behaviors. Surveys of the general public in Japan also demonstrated substantial gaps in knowledge of HIV/AIDS. Tweets from the social networking service X (formerly known as Twitter) have been studied to identify stigmas in other disorders but have not yet been used to study HIV stigma in Japan.

**Objective:**

The aim of the study is to examine the variations in stigma related to HIV over an extended period using tweets from X and to investigate the stigma toward people with HIV associated with various demographic segments.

**Methods:**

Japanese tweets from X related to HIV/AIDS were retrospectively collected; the phase 1 feasibility study collected tweets from 2011, 2014, and 2017, and the phase 2 analysis included tweets from each third year from 2011 through 2023. Individual tweets were labeled with the messages they conveyed (stigma and corresponding antistigma types included labels, marks, responsibility, peril, insults, and fear; tweets without stigma or antistigma messages were considered general education or neutral) along with demographic characteristics and locations; phase 1 results were used to develop a machine learning model to apply in phase 2. The labeled data from phase 2 were used to answer research questions concerning yearly changes in HIV stigma and proportions of stigma across population segments.

**Results:**

A total of 2,016,826 tweets related to HIV/AIDS were identified over the study period; 1,648,556 (81.7%) were from individual accounts, with the remainder from organizational accounts. In total, 574,687 (28.5%) tweets indicated stigma attitudes, while 1,119,852 (55.5%), 207,320 (10.3%), and 114,967 (5.7%) showed neutral, antistigma, or general education attitudes, respectively. Tweets including peril, fear, or insult comprised 502,134 (87.4%) of tweets with stigma. The greatest numbers of tweets were made by people in their 20s, whereas people in their 20s and 60s had the greatest proportions of tweets with stigma (n=9650, 35.3% and n=558, 34.5%, respectively). Peril and fear made up 5819 (60.3%) of stigma tweets from people in their 20s. The proportion of tweets with stigma (n=59,719, 20.5% in 2017) increased notably during the COVID-19 pandemic (n=217,512, 31.4% in 2020, and a similar n=175,647, 33.9% in 2023). Tweets from health care practitioners had 1.68 times the odds of having antistigma messages versus those from others.

**Conclusions:**

This study contributes to the understanding of HIV stigma in Japan and shows the usefulness of social media for studying stigma. The extent and type of HIV stigma changed from before to after the COVID-19 pandemic. These results can be used to develop future activities and educational programs to combat HIV-related stigma.

## Introduction

With the availability of effective antiretroviral treatment, mortality among people with HIV has decreased considerably; in Japan, the 10-year survival rate for people with HIV no longer differs significantly from that for the general population [1]. Further, modern antiretroviral therapy is able to suppress viremia sufficiently to prevent sexual transmission of HIV [2]. Yet, public perception may not necessarily reflect such progress. In the 2018 “Public Opinion Survey on HIV Infection and AIDS” of Japanese people randomly selected by the Cabinet Office, approximately 33% of respondents identified AIDS as a disease with no known cause, indicating that the public’s understanding of HIV/AIDS is still considerably insufficient [3]. Moreover, internet search data indicated a decrease in searches relating to HIV test centers in Japan over the course of the COVID-19 pandemic [4]. Despite advances in medical treatments and improved availability of information, the stigma associated with HIV infection continues to be a major barrier to prevention, testing, treatment, and care [5-8]. Perceived stigma may influence people with HIV to withdraw from social relationships to avoid potential discrimination [9], leading to social isolation and reduced opportunities for social support. In health care settings, stigma decreases medication adherence and reduces trust in health care practitioners (HCPs) among those experiencing HIV stigma [10]. The effects of HIV stigma are not limited to health care behaviors or social interaction but also contribute to an overall poor quality of life [11-13]. HIV stigma can lead to disease progression by delaying care, as shown by the United Nations Development Programme’s correlation between stigma and disease, and many of the people most susceptible to HIV already face stigma, prejudice, and discrimination in their daily lives. This puts them on the margins of society, where poverty and fear make it difficult for them to access health care and HIV services [14]. A study by Yu et al [15] also reported that the prevalence of anticipated stigma was significantly higher among people with HIV from East Asia (China, Japan, South Korea, and Taiwan) than those from outside Asia.

Stigma refers to negative attitudes, beliefs, and behaviors toward a group (eg, people with HIV) [16]. Stigma can manifest as various messages, eliciting message responses, and ultimately causing message effects, as defined by Smith’s stigma-communication model, which can be used to analyze tweets from X (formerly known as Twitter) [17]. A message refers to the intent of a tweet, such as a label intended to reinforce societal prejudices. A message response is a reaction or interaction generated by another user in relation to the initial message, such as accessing relevant social attitudes or stereotypes. Message effects relate to the impact and consequences of these messages and responses on individuals affected by HIV stigma, particularly the development of stigmatizing attitudes [18].

The usefulness of studying the content of X tweets has been reported in recent years [19], and content from X has been used in studies examining the stigma associated with mental illness, obesity, and other disorders [18,20,21]. Ireland et al [22] also showed an association between more frequent “action” tweets and lower HIV incidence in the United States. Japan has the second-highest number of X users across all countries, with approximately 70 million users [23]. However, there have been no studies using X to evaluate attitudes toward HIV in Japan. The sheer volume of posts on X makes it impractical for researchers to personally review a representative portion for analysis. Natural language processing models are increasingly used for health informatics purposes, as they offer the possibility of analyzing volumes of data beyond the scale of practical human labor, including social media output [24]. The adoption of the Bidirectional Encoder Representations From Transformers (BERT) approach allows for the pretraining of models before they are applied to broader tasks [25]. The purpose of this study was to clarify the reality of HIV stigma in Japan by analyzing tweets on the social networking service X.

## Methods

### Study Phases

The study was conducted in 2 phases. A feasibility study phase was conducted first to explore and contrast the different methodologies of analysis to better understand which approach best answers the research question associated with the study objectives. Then, the outcome of the feasibility study was used as a reference to perform an expanded study on a larger dataset obtained from mining tweets collected over a longer period.

### Research Objectives

The primary research objective was to examine the variations and transformations in stigma related to HIV over an extended period, and the secondary objective was to analyze the unique stigma associated with demographic segments, including age, sex, and geographic area groups; HCPs or non-HCPs; and individual or organizational accounts.

### Ethical Considerations

This study did not directly involve human participants or include any interventions but instead used publicly available tweets. Nonetheless, the investigator submitted this protocol to the independent research ethics committee Non-Profit Organization MINS (Tokyo, Japan; approval 240209) and ensured compliance with the research ethics principles outlined in the Declaration of Helsinki (7th revision, 2013). Before implementation, the investigator received and documented approval from the NPO MINS for any modifications made to the protocol. No informed consent was obtained to participate in this secondary analysis of existing data; X users agree to public privacy settings in the Terms and Agreements. Personal identifying information was limited to information the X users chose to make public. No compensation was provided for X users.

### Inclusion and Exclusion Criteria

The study referenced previous HIV/AIDS infodemiology papers [26-28] to create the initial list of search keywords for obtaining target tweets using the X application programming interface and to develop the methodology for selecting keywords. HIV, AIDS, and their Japanese synonyms were selected as the keywords. The list was intentionally kept simple to reduce the “noise” that can be generated by using X as a data source.

Certain stop words were selected to remove unrelated data, such as the omission of records with “猫” [cat] to remove unwanted feline immunodeficiency tweets; these stop words are listed in Table 1. Unrelated tweets were excluded, as were bot and spam tweets with no identifiable human input.

**Table 1:** Unrelated topics and corresponding keywords used to exclude tweets from the analysis of stigma from 2011 through 2023 in Japan.

Topic	Keywords
Band-aids	Band-aid
Cat	猫 (Cat) ねこ (Cat in Hiragana) にゃん (Cat’s crying sound) 飼い主 (Pet owner) 里親 (Foster parent) のら (Wild cat in Hiragana) ノラ (Wild cat in Katakana) 野良 (Wild cat in Kanji)
Chernobyl-aids	チェルノブイリ (Chernobyl)
Fish or guppy-aids	魚 (Fish) グッピー (Guppy) 匹 (Unit of counting fish)
Hearing	補聴器 (Hearing-aid machine) Hearing

### Data Collection

Tweet data and metadata, such as profile descriptions and follower counts, were obtained using the X application programming interface. Tweet data included the tweeter, the tweet itself, the time of the tweet, and the prefecture (Table 2). Demographic segmentation information was extracted from the profile to the extent possible, including age bracket, sex, prefecture, profile text, and profession (HCP or non-HCP). Tweet data and profile data were linked in a master dataset. Proportions of the data unsuitable for the analyses could be removed.

**Table 2:** Data collected about tweets for categorization in the analysis of stigma from 2011 through 2023 in Japan.

Collected field	Description
tweet_id	A unique combination of integers used to identify all tweets.
tweet_text	The UTF-8^a^ text of the tweet.
is_retweet	A Boolean (true or false) used to identify whether a tweet is original or retweeted. Retweeted tweets may be one of the following: quotation, retweet, or comment.
tweet_author	A unique combination of integers used to identify the author of the tweet. Forms a one-to-one relation with the author_id field.
conversation_id	The tweet_id of the original tweet of the conversation (which includes direct replies and replies of replies). Used to reconstruct a conversation or dialogue.
tweet_geo	The geolocation information of the tweet in text format. Available if the author of a tweet included a geolocation tag and NaN^b^ otherwise.
tweet_created_time	The UTC^c^ of tweet in ISO8601^d^ format.
referenced_tweets_id_list	A list including each tweet_id the associated tweet references. Only available if is_retweet is true, and NaN otherwise.
entities_expanded_url_list	A list of URL entities that have been parsed out of the tweet_text by X.
public_metrics_retweet_count	The integer retweet count of the associated tweet. A retweet occurs when a user shares another user’s tweet. Retweets do not include quotation tweets (retweets with a new comment).
public_metrics_reply_count	The integer reply count of the associated tweet. A reply occurs when a user comments under another user’s tweet.
public_metrics_like_count	The integer “like” count of the associated tweet. A like occurs when a user clicks the heart button of another user’s tweet.
public_metrics_quote_count	The integer quoted count of the associated tweet. A quotation occurs when a user tweets some original text upon referencing or citing another user’s tweet.
public_metrics_bookmark_count	The integer bookmarked count of the associated tweet. A bookmark occurs when a user saves another user’s tweet for later access.
public_metrics_impression_count	The integer impression count of the associated tweet. An impression occurs when a tweet becomes visible anywhere on a viewer’s screen.
author_metrics_followers_count	The integer accumulated follower count of the author of the associated tweet.
author_metrics_following_count	The integer accumulated following count of the author of the associated tweet.
author_metrics_tweet_count	The integer accumulated tweet count of the author of the associated tweet.
author_metrics_listed_count	The integer accumulated list count of the author of the associated tweet. A list indicates a categorization or grouping of X users into specific subject matters.
author_metrics_like_count	The integer accumulated “like” count of the author of the associated tweet.
author_description	The UTF-8 profile text of an author. Also known as a bio.
author_id	A unique combination of integers used to identify the author of the tweet. Forms a one-to-one relation with the tweet_author field.
author_name	The UTF-8 text name of the author as defined in the profile.
author_username	The UTF-8 text screen name, handle, or alias. Unique to each author but subject to change.
author_created_time	The UTC of user account creation in ISO8601 format.
author_location	The UTF-8 text location of the author. NaN if the author does not provide a text value.

^a^UTF: unicode transformation format.

^b^NaN: not a number.

^c^UTC: coordinated universal time.

^d^ISO: International Organization for Standardization.

Japanese tweet data were available from April 2008 onward, when X was launched in Japan. However, over the first 3 years, there was little use of the platform in Japan. For this study, 2011 was set as a new initial year, as the use of X in Japan is reported to have substantially increased after the 2011 earthquake off the Pacific Coast of Tohoku [29,30].

### Annotation

#### Message Category

Labels were applied to each tweet so the analysis result could be grouped and observed based on the stigma message and socioeconomic or geographic segments. Tweets were classified into one of the message categories: stigma (labels, marks, responsibility, peril, insults, and fear), corresponding antistigma (messages attempting to combat or rectify labels, marks, responsibility, peril, insults, and fear), general education, or neutral, as shown in the illustrative examples in Table 3. Based on the categories outlined by Brown et al [18] for investigation using Twitter, an additional stigmatic theme called fear was identified, as some tweets expressed fear toward HIV/AIDS without necessarily inciting social peril. General education was separated from antistigma as an independent category; this was used for tweets that may have provided useful information for people with HIV or the general population while not necessarily directly addressing or combating stigma. The initial categories were examined through the labeling processes, and their definitions (the codebook) were updated or elaborated as needed.

A portion of tweets was manually labeled by a group of coders to serve as the teaching data for supervised learning in both the primary and secondary analyses. The methodology was established in accordance with previous research [31] and included sampling and distribution of up to 10 people for labeling. To reduce bias, a small sample of tweets (approximately 1500 tweets per labeler) distributed to the labelers received overlapping treatment, thereby affording them a chance to be labeled by at least 2 people. This promoted consistency in labeling across different labelers. To establish further rigor, the coding team lead was designated in advance and tasked to maintain an audit trail of the manual coding processes. A guiding codebook was developed through interaction with the team lead to ensure agreement. Any contradictory interpretations were resolved through reflective practice and systematic consultations with the entire team. The team could decide to review auxiliary information, such as linked websites and tweets immediately before and after the extracted tweets, in a preagreed manner. The discrepancy resolution process was repeated until 80% of the distributed data were labeled consistently across the annotators. After an initial round of labeling based on randomly sampled posts, categories with low frequency (eg, antistigma) were chosen for additional sampling. Generative artificial intelligence (ChatGPT) with message definitions included in the prompt was used to screen the posts for annotation. However, all posts were ultimately manually annotated for machine learning training purposes.

**Table 3:** Stigma subclassification definitions used from analysis of stigma in tweets from 2011 through 2023 in Japan^a^.

Types of stigma	Definition	Example tweets
Label	Labels are othering terms used to refer to a stigmatized group. Labels may cause people to consider the stigmatized persons as a distinct group, highlight group differences, or promote stereotypes by highlighting particular characteristics associated with the PSHCD^b^.	“I don’t want to be with homosexuals because I don’t want to get AIDS.” “Black people have quite a lot of HIV infections.”
Marks	Marks refer to potentially stigmatizing images, describing ways to identify members of a stigmatized group. To be most effective, marks may be visible physical features, perceived as disgusting, recognized quickly, and remembered.	“I was chatting with an infectious disease doctor and the topic of AIDS came up. Apparently, there are HIV specialists who can tell if someone has HIV just by a glance. They say things like, ‘I mean, even the way they open the door seems suggestive.’ I wonder if there are cases where a person mimics the behavior or habits of an HIV-negative person and unknowingly contracts HIV.” “Kaposi’s sarcoma all over the face and body. If you see it like that, it’s AIDS.”
Responsibility	Responsibility messages attribute blame or fault to an individual or group because of their experiences of the target PSHCD. Such messages may ascribe personal responsibility for the origin, persistence, or severity of the target PSHCD.	“AIDS, huh ... This one is all about personal responsibility. If you want to prevent infection, you have to take precautions yourself. And you really shouldn’t go to brothels in the first place. The risk is naturally higher for those who frequent such places compared to the average person. You just have to understand that and make your choices accordingly.” “HIV, because homosexuals do whatever the hell they want to do to each other.”
Peril	Peril is content that describes the physical or social threat to a community’s effective functioning. To be most effective, the peril may be painful, deadly, and socially taboo.	“I heard that more and more foreigners with AIDS virus are coming to Japan and prostituting themselves in Japan. Be careful, sex workers!” “A 28-year-old woman infected with HIV is having sex with more than 300 people to get revenge on a man, and she can’t be arrested, so she’s on the loose, Welcome to the AIDS world.”
Insults	Insulting stigma includes messages that are devaluing, derogatory, or abusive toward those living with potentially stigmatized health conditions and disorders. The life of an individual or group may be devalued by content that suggests someone’s status, character, credibility, or value may be lessened because of their experiences of the target PSHCD. Derogatory language refers to communications that use terms associated with the target PSHCD in a disparaging or disrespectful manner. This includes referencing terms associated with the target PSHCD outside of a health context. Abusive communication is content that involves web-based harassment by direct or indirect reference to the target PSHCD.	“Record number of AIDS cases, huh. But the rise in new infections is even more worrying. Increases among people in their 40s and 60s ... Gross old folks causing problems.” “AIDS lectures, what a pain in the ass. Sex, gays, etc., really annoying.”
Fear	Fear is the demonstration of baseless personal anxiety toward HIV/AIDS in general or phobia of the people involved. It also involves distrust and discomfort over a situation that is, in fact, within one’s control or already contained.	“I’m scared of AIDS and hemorrhoids, so I don’t want to do the real fuck.” “I’m afraid I’ll get HIV if I shake their hand.”

^a^Based on Brown et al [18].

^b^PSHCD: potentially stigmatized health condition or disorder.

#### Socioeconomic or Geographic Segments

Data segmentation was also performed using a tagging task to identify the age, sex, and other information regarding the user (Table 4), using the user profile text as input, to address the secondary objective of examining HIV stigma associated with various demographic segments. The age segment was divided into brackets of 10 years. The segment of the geographic area was used instead of the prefecture due to a scarcity of tweets with prefecture label; the geographic area segment was divided into metropolis, metropolis peripheral city, medium-sized city, or others. The sex and HCP or non-HCP segments were categorized using binary labels (sex other than male or female could not be included due to lack of representation). Furthermore, the representation and media segments were given particular attention. Subgroup analysis was used to delve into the segmentation of stigmatizing messages. A more detailed examination was performed, if tweet volume permitted, to explore the interplay between different segment combinations or investigate the temporal dynamics of these segments.

**Table 4:** Segments of categorization from analysis of stigma in tweets from 2011 through 2023 in Japan.

Category and segment			Label
**Binary label**			
	Sex of tweeter	Male or female^a^	
	Profession of tweeter	Health care practitioner or not health care practitioner	
	Representation of tweeter	Individual or organization (media or general)	
**Multilabel**			
	Age of tweeter	10-19, 20-29, 30-39, 40-49, 50-59, or 60+ years	
	Prefecture of tweeter^b^	Tokyo, Kyoto, or any of the other 47 prefectures of Japan	

^a^Sex other than male or female was planned for inclusion but could not be included due to lack of representation.

^b^A segmentation category of “area” was introduced to gain further insight into stigma levels between urban versus nonurban environments, as not enough tweets with prefecture labels were available, and the results were not necessarily reliable at the prefecture level.

### Modeling

#### Message Category

To fulfill the primary objective of classifying HIV stigma type or message on tweets, the research included “stigma modeling” using a BERT-based large language model [32]. Transfer learning from Hugging Face’s publicly available large language model was used to fine-tune this classification task [33]. A stigma model and an antistigma model were developed (each considering the categories of fear, insult, label, etc). The performance of the models was evaluated using the area under the curve (AUC) based on the receiver operating characteristic curve. An AUC of 1 indicates a perfect model, whereas an AUC of 0.5 signifies a model that is no better than random chance.

#### Socioeconomic or Geographic Segments

The methodology used for the segmentation analysis involved constructing models tasked with categorizing the data into specific segments. Models (machine learning or rule-based) were built to label each of the segments in the segmentation step for the secondary analyses (Table 5). In addition to the BERT model used for assigning message categories, there was a need for models that are quick to train and lightweight for the purpose of profiling demographics. A random forest model, known for its efficiency and robustness when examining unbalanced data [34], was used with MeCab tokenization and term frequency-inverse document frequency vectorization to assign demographic segments, including sex, health care profession, and representation. To enhance accuracy, label-identifying keywords and characters were used as separate predictors. For instance, Chinese characters corresponding to “father,” “male,” “uncle,” etc, in English, were chosen for the sex model. We decided to develop simple rule-based models to categorize location and age brackets. A pattern of age or birthday or birth year writing was first identified by examining the profiles, and those that matched the pattern were extracted. For example, numerical values preceding characters corresponding to “year old” and “generation” were extracted. For locations, all possible iterations of a prefecture name and its major city in Chinese characters, Japanese hiragana, katakana, and Roman alphabets were extracted.

The performance of all the trained models was evaluated using the AUC based on the receiver operating characteristic curve. For multiclass models, the one-versus-rest scheme was used. The results were cross-validated to ensure consistent performance across the models. The statistics of the results from rule-based models were evaluated against publicly available information, such as Japanese demography across prefectures, to identify potential bias and fine-tune the rules.

All models were based on the profiles and only the tweets with the defined HIV-related keywords [35]. Pictures, post histories, and other interactions on social media, such as likes, follows, and followers, were not used for this study.

**Table 5:** Models used to classify tweets in the analysis of stigma from 2011 through 2023 in Japan.

Task	Model	Input	Processing
Stigma message	Japanese BERT^a^	“tweet_text”	BERT Japanese tokenization
Sex	Random forest	“author_name” “author_description”	MeCab tokenization Tf-idf^b^ vectorization Add male flag if either input contains sex-identifying keywords: 男 (Male) 父 (Father) おじさん (Uncle) おっさん (Uncle) Add female flag if either input contains sex-identifying keywords: 女 (Female) 母 (Mother) 婦 (Woman)
Health care practitioner	Random forest	“author_name” “author_description”	MeCab tokenization Tf-idf vectorization Add medical flag if either input contains keywords: 医 (Medical) 介護 (Nursing care) 福祉 (Welfare) 病院 (Hospital)
Representation	Random forest	“author_name” “author_description” “author_metrics_followers_count”	MeCab tokenization Tf-idf vectorization Add media flag if author_name or author_description contains keywords: ニュース (News) News 新聞 (Newspaper) Add group flag if author_name or author_description contains keywords: 部 (Department) 会 (Association)

^a^BERT: Bidirectional Encoder Representations From Transformers.

^b^Tf-idf: term frequency-inverse document frequency.

### Data Analysis

The next step in the research process involved conducting quantitative analyses. The frequency of message choice was calculated for each message-choice category (labels, marks, responsibility, peril, insult, and fear for stigma and corresponding categories for antistigma). The yearly frequency of each message-choice category was aggregated between 2011 and 2023.

Time-series charts were used to plot the stigma and antistigma counts and proportions of total tweets, providing a clear visualization of the yearly change in public stigma. Subgroup analyses were conducted to investigate the potential intersectionality of stigma levels among the assessed segments. The proportion of stigmatizing and antistigmatizing tweets was calculated for each pair of compared segments (eg, male vs female). Fisher exact test with Holm’s method was performed to assess any differences across the segmented subgroups. Multivariate logistic regression was used to examine the characteristics associated with stigma.

## Results

### Characteristics of Users Posting Tweets

**Figure 1:**
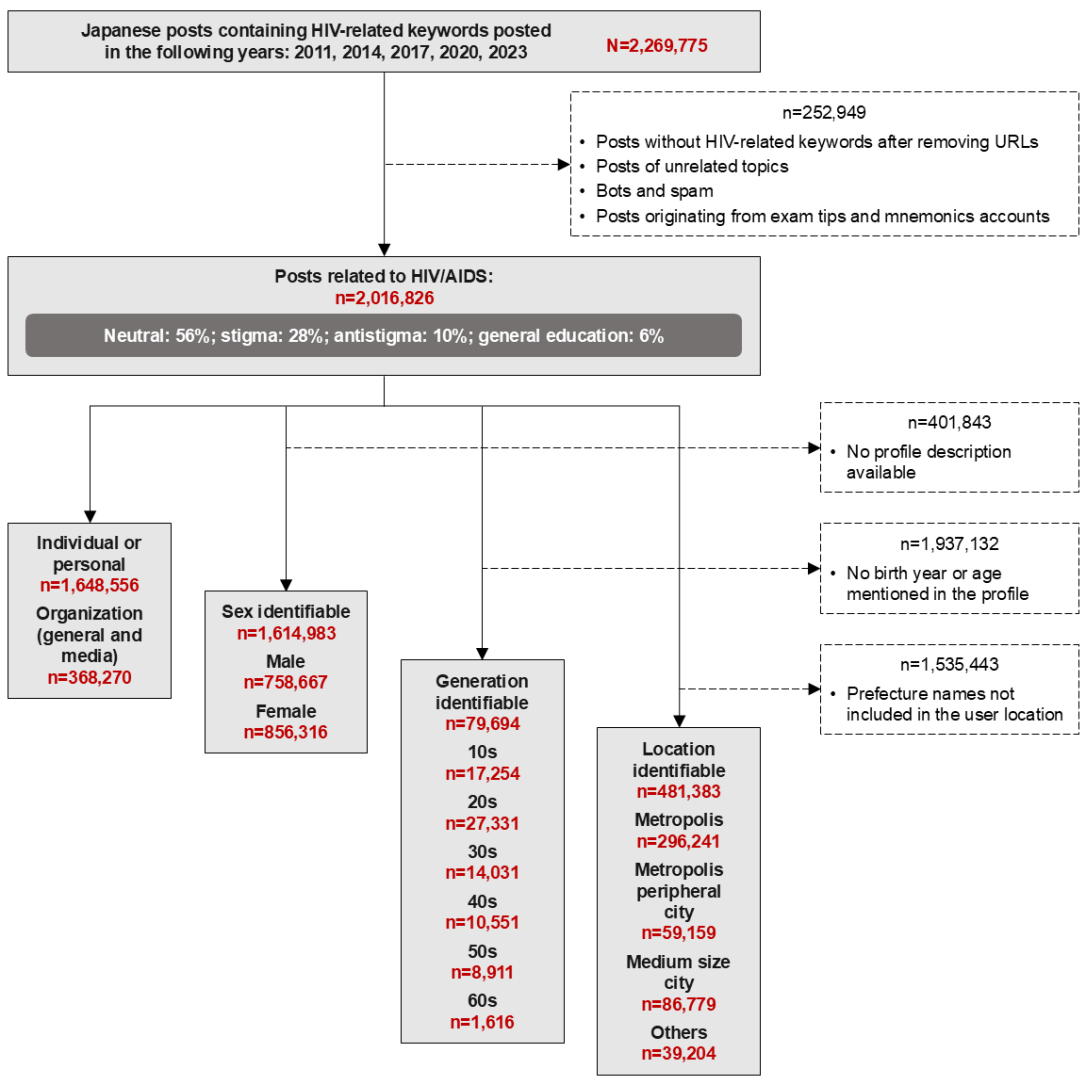
Quantification of tweets identified in the analysis of stigma from 2011 through 2023 in Japan.

A total of 2,016,826 tweets related to HIV/AIDS were identified (Figure 1). Among these, sex could be identified for 1,614,983 (80.1%), age group could be identified for 79,694 (4%), and prefecture could be identified for 481,383 (23.9%).

Characteristics of X users included in the study are shown in Table 6. Of 2,016,826 tweets, 1,648,556 (81.7%) were from individuals; the remainder came from organizations. Among tweets from individuals, 1,610,060 (97.7%) were from non-HCPs; among people whose age group could be identified, more tweets were from people in their 20s compared with any other decade (27,331/79,694, 34.3%); and among those tweets from individuals whose location could be identified (n=481,383), the majority came from people in a metropolis (n=296,241, 61.5%).

**Table 6:** Characteristics of tweets and X users in the analysis of stigma from 2011 through 2023 in Japan.

Attributes		Total, n (%)	Neutral, n (%)	Stigma, n (%)	Antistigma, n (%)	General education, n (%)
**Representation**		2,016,826 (100)	1,119,852 (55.5)	574,687 (28.5)	207,320 (10.3)	114,967 (5.7)
	Individual	1,648,556 (81.7)	898,186 (54.5)	513,961 (31.2)	177,421 (10.8)	58,988 (3.6)
	Organization: general	344,801 (17.1)	204,765 (59.4)	56,258 (16.3)	28,291 (8.2)	55,487 (16.1)
	Organization: media	23,469 (1.2)	16,901 (72)	4468 (19)	1608 (6.9)	492 (2.1)
**Sex**		1,614,983 (100)	877,197 (54.3)	506,335 (31.4)	173,796 (10.8)	57,655 (3.6)
	Male	758,667 (47)	401,123 (52.9)	254,263 (33.5)	75,960 (10)	27,321 (3.6)
	Female	856,316 (53)	476,074 (55.6)	252,072 (29.4)	97,836 (11.4)	30,334 (3.5)
**HCP^a^ or non-HCP**		1,648,556 (100)	898,186 (54.5)	513,961 (31.2)	177,421 (10.8)	58,988 (3.6)
	HCP	38,496 (2.3)	21,790 (56.6)	8723 (22.7)	6421 (16.7)	1562 (4.1)
	Non-HCP	1,610,060 (97.7)	876,396 (54.4)	505,238 (31.4)	171,000 (10.6)	57,426 (3.6)
**Age group**		79,694 (100)	40,172 (50.4)	22,986 (28.8)	10,053 (12.6)	6483 (8.1)
	10s	17,254 (21.7)	7699 (44.6)	3774 (21.9)	1338 (7.8)	4443 (25.8)
	20s	27,331 (34.3)	13,707 (50.2)	9650 (35.3)	3249 (11.9)	725 (2.7)
	30s	14,031 (17.6)	7259 (51.7)	4052 (28.9)	2179 (15.5)	541 (3.9)
	40s	10,551 (13.2)	5789 (54.9)	2654 (25.2)	1806 (17.1)	302 (2.9)
	50s	8911 (11.2)	4908 (55.1)	2298 (25.8)	1309 (14.7)	396 (4.4)
	60s	1616 (2)	810 (50.1)	558 (34.5)	172 (10.6)	76 (4.7)
**Area**		481,383 (100)	299,682 (62.3)	115,652 (24)	53,709 (11.2)	12,340 (2.6)
	Metropolis	296,241 (61.5)	182,234 (61.5)	72,006 (24.3)	34,775 (11.7)	7226 (2.4)
	Metropolis peripheral city	59,159 (12.3)	35,173 (59.5)	15,685 (26.5)	6858 (11.6)	1443 (2.4)
	Medium-sized city	86,779 (18)	57,866 (66.7)	18,778 (21.6)	8010 (9.2)	2125 (2.4)
	Others	39,204 (8.1)	24,409 (62.3)	9183 (23.4)	4066 (10.4)	1546 (3.9)

^a^HCP: health care practitioner.

### Characteristics of Tweets

**Figure 2:**
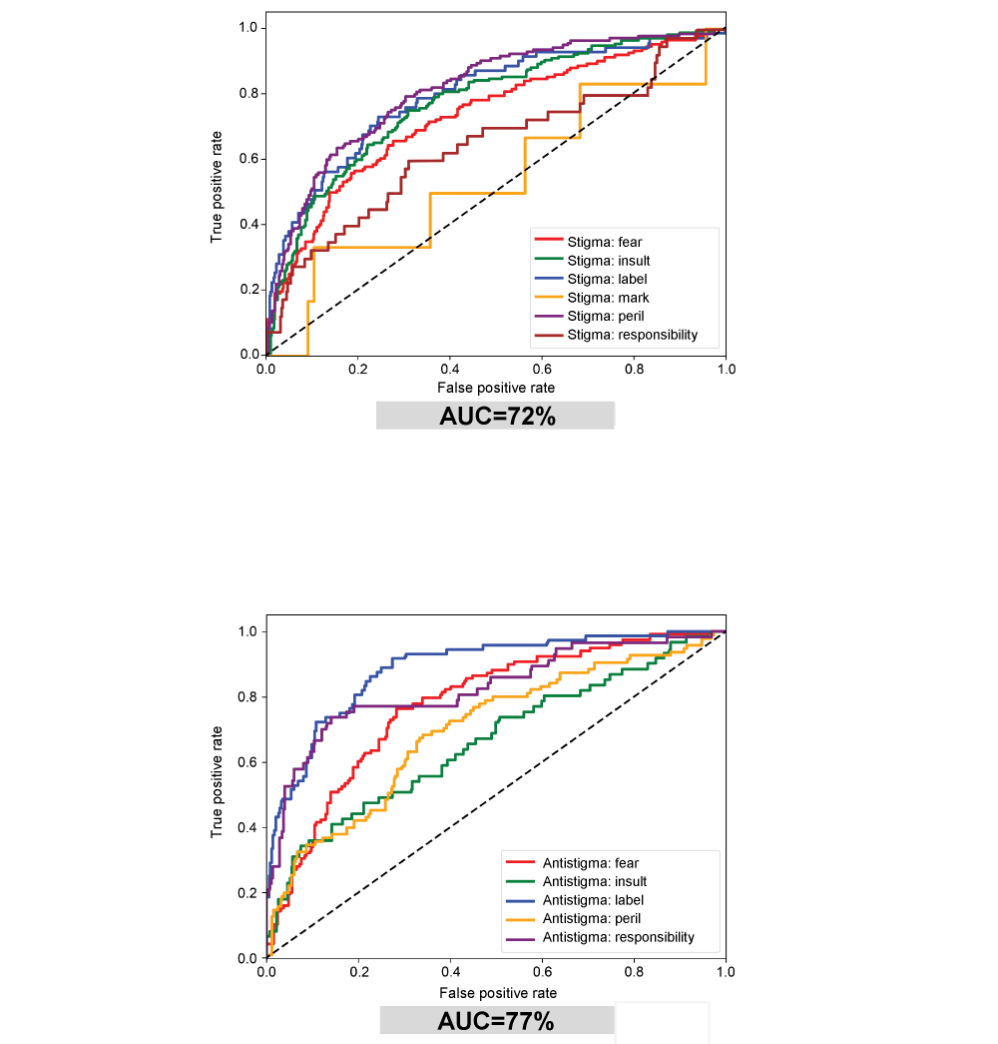
Receiver operating characteristic curves showing the evaluation of (top panel) stigma model and (bottom panel) antistigma model used in the analysis of tweets from 2011 through 2023 in Japan. The performance of the machine learning models was found to have an AUC of 0.72 for the stigma model and 0.77 for the antistigma model, indicating moderate performance. AUC: area under the curve.

**Figure 3:**
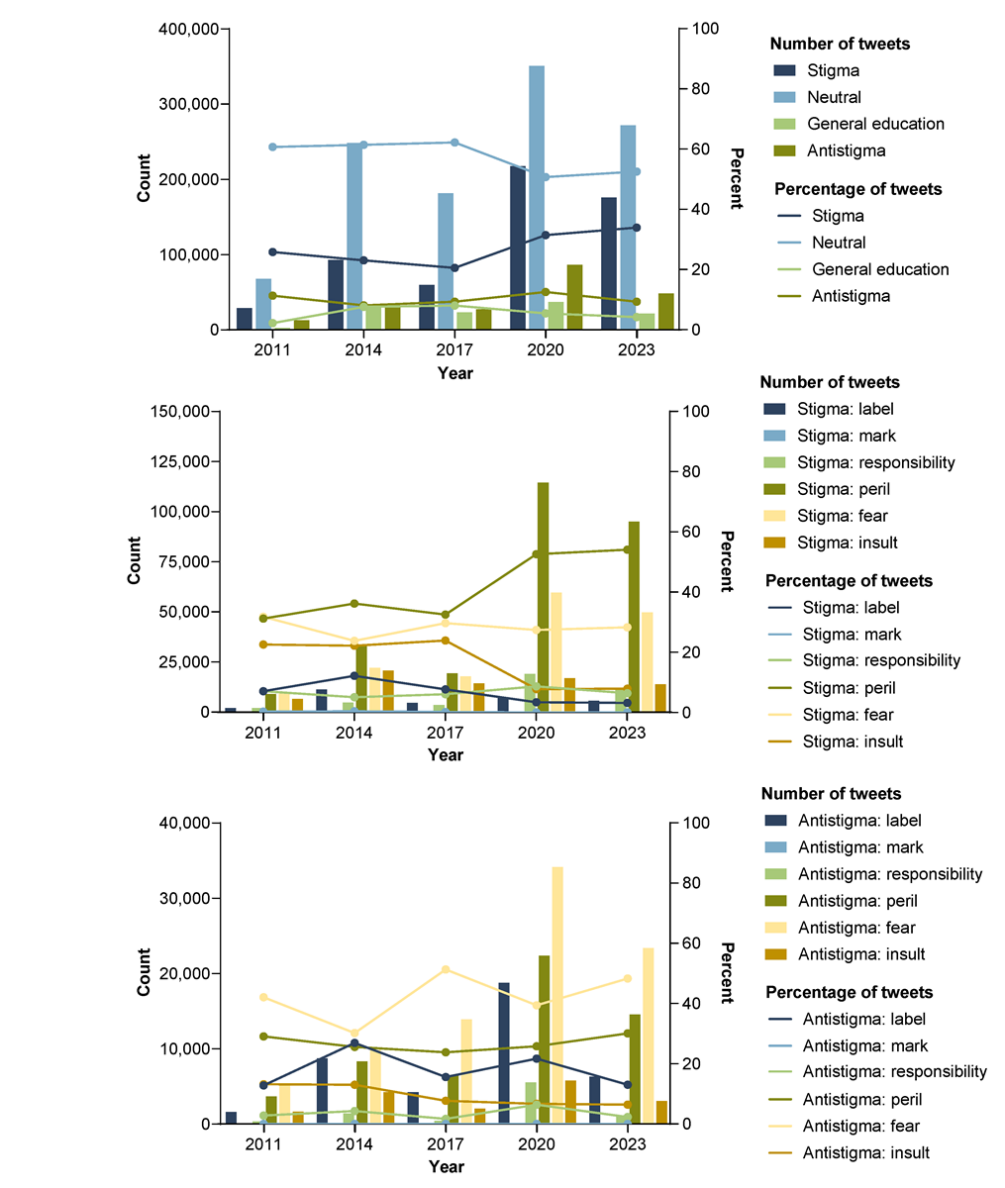
Message classification trends for analysis of tweets from 2011 through 2023 in Japan: (top panel) number and percentage of tweets with stigma, neutral, general education, or antistigma labels; (middle panel) number and percentage of tweets with different types of stigma; and (bottom panel) number and percentage of tweets with type of antistigma label. Toward the end of 2023, the proportion of tweets with stigma substantially exceeded the pre–COVID-19 pandemic level.

In terms of attitude, 1,119,852 (55.5%) of all tweets were neutral versus 574,687 (28.5%), 207,320 (10.3%), and 114,967 (5.7%) with stigma, antistigma, or general education attitudes, respectively (Table 6). The performance of the machine learning models was found as an AUC of 0.72 for the stigma model and 0.77 for the antistigma model (Figure 2), indicating moderate performance. The stigma model had a slightly lower performance due to the lack of training data for the mark category (less than 1% incidence rate), though it was within the acceptable range. In the antistigma model, the mark category was excluded due to its extremely low incident rate. The additional model constructed to classify tweets that were considered neither stigma nor antistigma into general education, irrelevant, or neutral categories had an AUC of 0.92. The proportion of tweets with stigma, 20.5% (n=59,719) in 2017, increased significantly during the COVID-19 pandemic; even toward the end of 2023, the proportion of tweets with stigma was 33.9% (n=175,647), substantially exceeding the pre–COVID-19 pandemic level (Figure 3). Until 2020, absolute numbers rose, the stigma ratio decreased, and the general education ratio increased. After the onset of the COVID-19 pandemic in 2020, except for only a slight increase in general education, all classifications saw a large surge in numbers. By 2023, numbers decreased from 2020, but the stigma ratio remained higher than in 2011. When comparing data before and after the COVID-19 pandemic, stigma rates increased for peril and decreased for insult. In 2020, the number of tweets including peril, fear, and responsibility surged, corresponding with the COVID-19 pandemic. Changes in mentions of other stigma were relatively constant after peaking in 2014, although label and insult decreased in absolute numbers relative to their 2014 peak. There were more tweets with stigma messages than antistigma messages at each time point, and the relative frequency of antistigma tweets, compared to stigma tweets, remained relatively consistent before and after the pandemic. After 2020, the total tweet count surged; it may be the case that antistigma tweets increased in response to the increase in stigma messages during the COVID-19 pandemic. On the other hand, within the breakdown of antistigma types, there was no notable trend before and after the pandemic, and proportions were relatively constant compared to the stigma breakdown ratio.

In terms of stigma subcategory, peril (n=271,614, 47.3%) was the most prevalent, followed by fear (n=158,328, 27.6%) and insult (n=72,192, 12.6%), but the proportion of stigma categories varied depending on the attributes of the individuals posting the tweets (Table 7). The proportion of tweets including fear was greater among HCPs than among non-HCPs (n=2772, 31.8% vs n=139,502, 27.6%). Among X users in their 20s, 881 (9.1%), 7 (0.1%), 1678 (17.4%), 2095 (21.7%), 4724 (38.6%), and 1265 (13.1%) tweets with stigma included label, mark, responsibility, peril, fear, and insult, respectively, versus 34 (6.1%), 1 (0.2%), 26 (4.7%), 264 (47.3%), 205 (36.7%), and 28 (5%) for X users in their 60s.

**Table 7:** Stigma and attribute classification from analysis of stigma in tweets from 2011 through 2023 in Japan.

Attribute		Stigma total, n (%)	Label, n (%)	Mark, n (%)	Responsibility, n (%)	Peril, n (%)	Fear, n (%)	Insult, n (%)
**Representation**		574,687 (100)	31,124 (5.4)	733 (0.1)	40,696 (7.1)	271,614 (47.3)	158,328 (27.6)	72,192 (12.6)
	Individual	513,961 (89.4)	26,070 (5.1)	652 (0.1)	38,111 (7.4)	238,103 (46.3)	142,274 (27.7)	68,751 (13.4)
	Organization: general	56,258 (9.8)	4656 (8.3)	50 (0.1)	2410 (4.3)	31,093 (55.3)	15,148 (26.9)	2901 (5.2)
	Organization: media	4468 (0.8)	398 (8.9)	31 (0.7)	175 (3.9)	2418 (54.1)	906 (20.3)	540 (12.1)
**Sex**		506,335 (100)	25,986 (5.1)	651 (0.1)	37,983 (7.5)	236,012 (46.6)	139,777 (27.6)	65,926 (13)
	Female	254,263 (50.2)	12,793 (5)	346 (0.1)	18,212 (7.2)	117,528 (46.2)	65,952 (25.9)	39,432 (15.5)
	Male	252,072 (49.8)	13,193 (5.2)	305 (0.1)	19,771 (7.8)	118,484 (47)	73,825 (29.3)	26,494 (10.5)
**HCP^a^ or non-HCP**		513,961 (100)	26,070 (5.1)	652 (0.1)	38,111 (7.4)	238,103 (46.3)	142,274 (27.7)	68,751 (13.4)
	HCP	8723 (1.7)	419 (4.8)	17 (0.2)	507 (5.8)	4236 (48.6)	2772 (31.8)	772 (8.9)
	Non-HCP	505,238 (98.3)	25,651 (5.1)	635 (0.1)	37,604 (7.4)	233,867 (46.3)	139,502 (27.6)	67,979 (13.5)
**Generation**		22,986 (100)	1777 (7.7)	26 (0.1)	2869 (12.5)	7196 (31.3)	8220 (35.8)	2898 (12.6)
	10s	3774 (16.4)	220 (5.8)	3 (0.1)	294 (7.8)	1450 (38.4)	1172 (31.1)	635 (16.8)
	20s	9650 (42)	881 (9.1)	7 (0.1)	1678 (17.4)	2095 (21.7)	3724 (38.6)	1265 (13.1)
	30s	4052 (17.6)	327 (8.1)	8 (0.2)	482 (11.9)	1178 (29.1)	1555 (38.4)	502 (12.4)
	40s	2654 (11.5)	191 (7.2)	2 (0.1)	213 (8)	1119 (42.2)	877 (33)	252 (9.5)
	50s	2298 (10)	124 (5.4)	5 (0.2)	176 (7.7)	1090 (47.4)	687 (29.9)	216 (9.4)
	60s	558 (2.4)	34 (6.1)	1 (0.2)	26 (4.7)	264 (47.3)	205 (36.7)	28 (5)
**Area**		115,652 (100)	5713 (4.9)	193 (0.2)	7408 (6.4)	57,813 (50)	32,811 (28.4)	11,714 (10.1)
	Metropolis	72,006 (62.3)	3518 (4.9)	134 (0.2)	4639 (6.4)	35,540 (49.4)	20,832 (28.9)	7343 (10.2)
	Metropolis peripheral city	15,685 (13.6)	756 (4.8)	27 (0.2)	990 (6.3)	7973 (50.8)	4365 (27.8)	1574 (10)
	Medium-sized city	18,778 (16.2)	908 (4.8)	26 (0.1)	1155 (6.2)	9753 (51.9)	5151 (27.4)	1785 (9.5)
	Others	9183 (7.9)	531 (5.8)	6 (0.1)	624 (6.8)	4547 (49.5)	2463 (26.8)	1012 (11)

^a^HCP: health care practitioner.

### Characteristics Associated With Stigma

**Figure 4:**
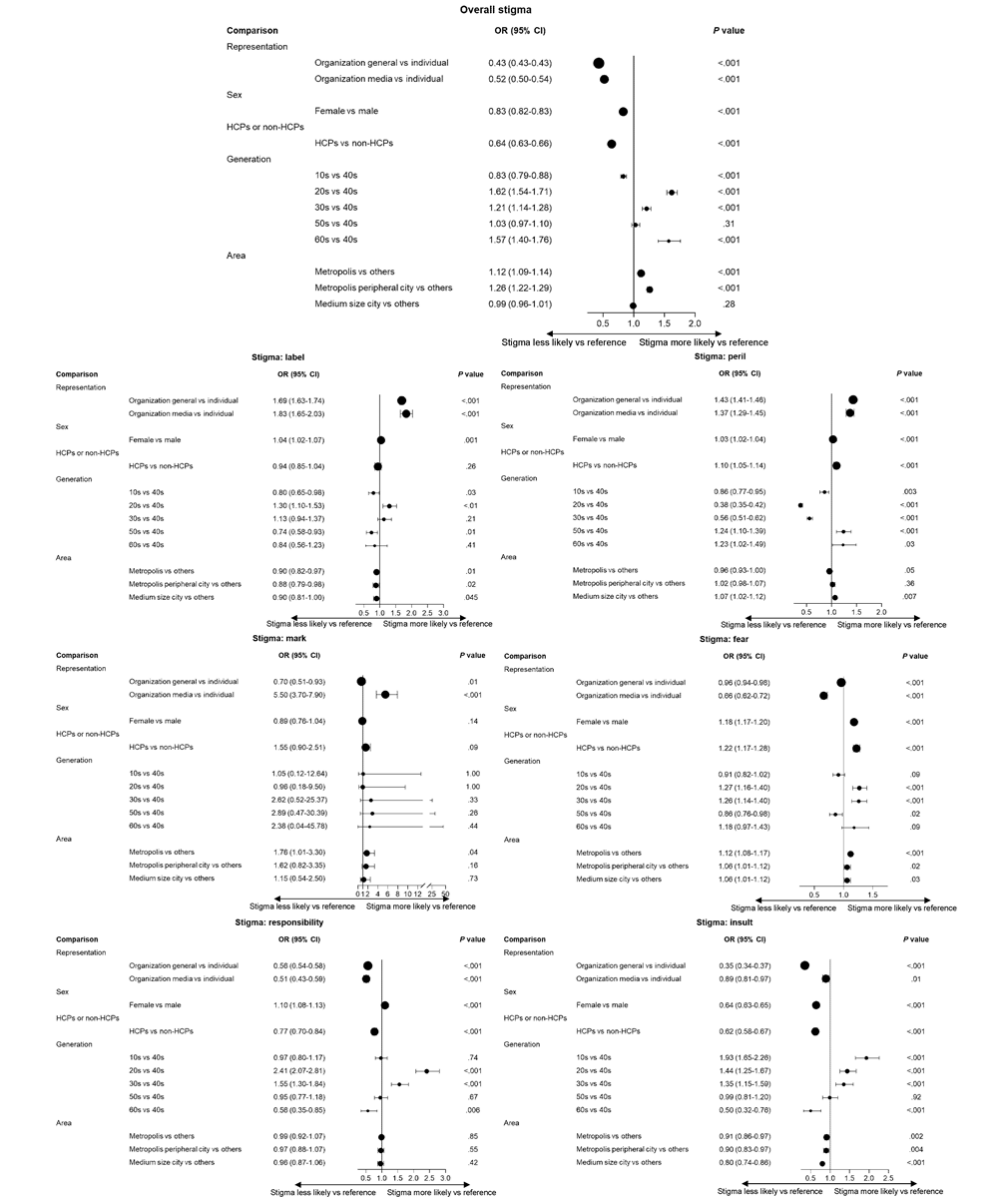
Characteristics associated with stigma messages in analysis of tweets from 2011 through 2023 in Japan: (top center) overall, (top of left column) label, (middle of left column) mark, (bottom of left column) responsibility, (top of right column) peril, (middle of right column) fear, and (bottom of right column) insult. HCP: health care practitioner; OR: odds ratio.

Models constructed to classify sex, individuals versus organizations, and HCPs versus non-HCPs had AUCs of 0.78, 0.89, and 0.94, respectively (models for age brackets and location were rule-based, and no AUCs applied). Figure 4 shows characteristics associated with a stigma message. Tweets from organizations were less likely than tweets from individuals to be associated with stigma (general organizations: odds ratio [OR] 0.43, 95% CI 0.43-0.43 and media organizations: OR 0.52, 95% CI 0.50-0.54 vs individuals). Tweets from people in their 20s (OR 1.62, 95% CI 1.54-1.71), 30s (OR 1.21, 95% CI 1.14-1.28), or 60s (OR 1.57, 95% CI 1.40-1.76) were all more likely to bear a stigma message versus tweets from people in their 40s. Tweets from organizations were more likely to be associated with the label stigma than were tweets from individuals (general organizations: OR 1.69, 95% CI 1.63-1.74 and media organizations: OR 1.83, 95% CI 1.65-2.03 vs individuals). Tweets from people in their 20s (OR 2.41, 95% CI 2.07-2.81) and 30s (OR 1.55, 95% CI 1.30-1.84) were more likely than tweets from people in their 40s to bear responsibility stigma. Tweets from organizations were more likely to bear the stigma of peril (general organizations: OR 1.43, 95% CI 1.41-1.46 and media organizations: OR 1.37, 95% CI 1.29-1.45 vs individuals). Tweets from organizations were less likely to be associated with fear stigma versus those from individuals (general organizations: OR 0.96, 95% CI 0.94-0.98 and media organizations: OR 0.66, 95% CI 0.62-0.72 vs individuals). Tweets from people in their 20s (OR 1.27, 95% CI 1.16-1.40), 30s (OR 1.26, 95% CI 1.14-1.40), and 60s (OR 1.18, 95% CI 0.97-1.43) were more likely to be associated with fear stigma than those from people in their 40s. Tweets from people in a metropolis or the peripheral cities of a metropolis (OR 1.12, 95% CI 1.08-1.17) were more likely to bear fear stigma than tweets from people in other locations (OR 1.06, 95% CI 1.01-1.12). Tweets from people in their teens (OR 1.93, 95% CI 1.65-2.26), 20s (OR 1.44, 95% CI 1.25-1.67), and 30s (OR 1.35, 95% CI 1.15-1.59) were more likely to bear insult stigma than tweets from people in their 40s.

**Figure 5:**
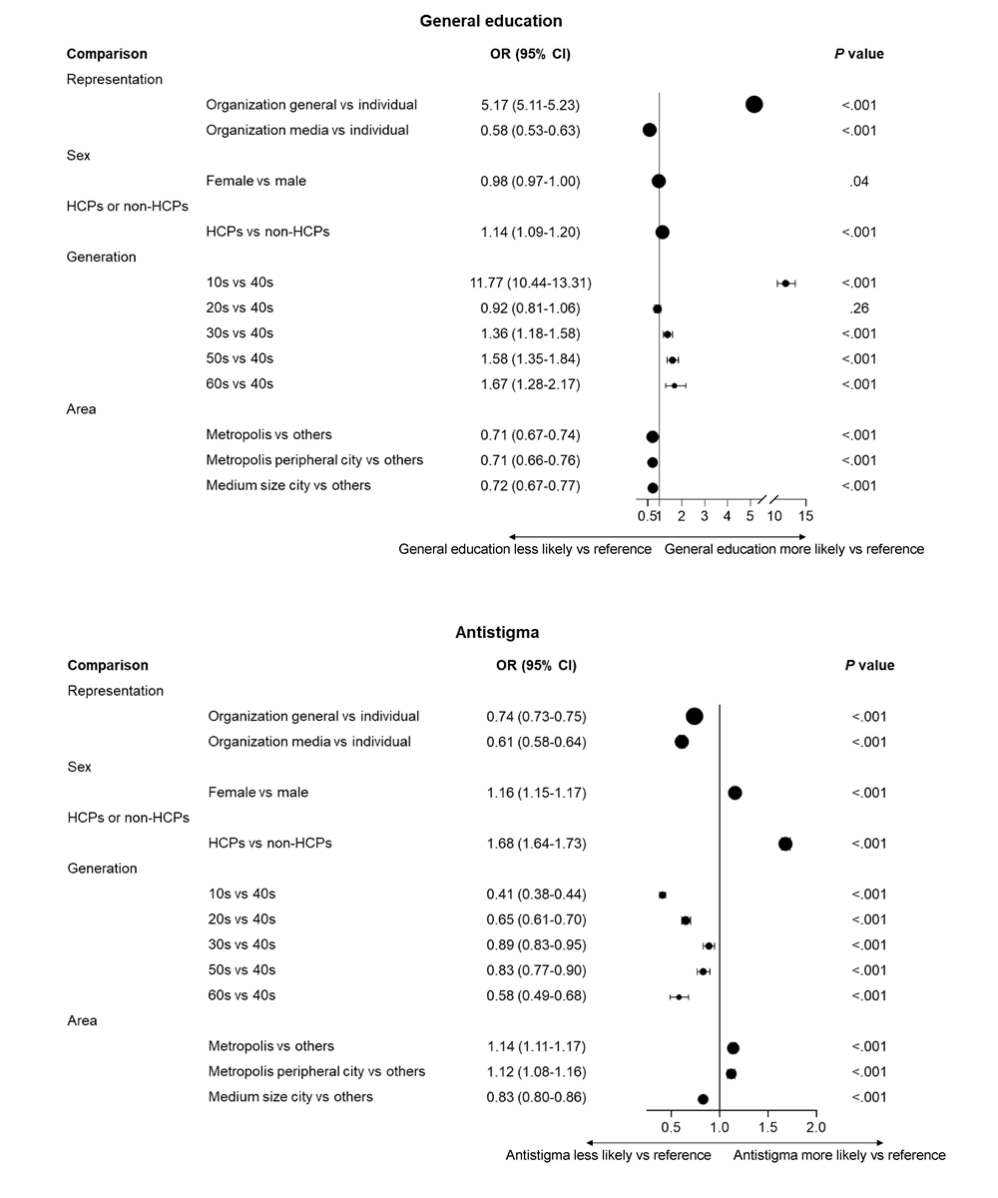
Characteristics associated with (top) general education and (bottom) antistigma in the analysis of tweets from 2011 through 2023 in Japan. HCP: health care practitioner; OR: odds ratio.

Tweets from general organizations were more likely than tweets from individuals to have a general education perspective (OR 5.17, 95% CI 5.11-5.23), whereas tweets from media organizations were less likely than tweets from individuals to have a general education perspective (OR 0.58, 95% CI 0.53-0.63; Figure 5). Tweets from HCPs were more likely than tweets from non-HCPs to have an antistigma perspective (OR 1.68, 95% CI 1.64-1.73), whereas tweets from people in a metropolis were more likely than tweets from people in other areas to have an antistigma perspective (OR 1.14, 95% CI 1.11-1.17).

## Discussion

### Principal Findings

In this retrospective study of tweets from Japan mentioning HIV/AIDS from 2011 through 2023 processed using machine learning, the greatest proportion of tweets were neutral; however, there were more tweets with stigma messages than antistigma messages. The tweets were overwhelmingly from individuals in metropolitan areas, which is consistent with expectations for social media. Disappointingly, the number of tweets with HIV-related stigma rose over the course of the study along with the increasing number of X users in Japan. The details identified in this study of population segments most likely to express stigma and the type of stigma expressed can help inform more effective educational activities in the future.

HIV-related tweets were predominantly posted by people in their 20s, indicating that young people may be more concerned about sexually transmitted infections such as HIV than are older people; however, it should be noted that X is a social networking site that may attract young people. Stigma increased during the COVID-19 pandemic (2020 vs 2011), including the percentage of tweets with the peril label. Conspiracy theories such as HIV being contained in the COVID-19 vaccine were spread on social networking sites during this period [36,37], and these tweets could be a possible reason for the increase in HIV-related tweets.

In tweets from people in their 20s and those in their 60s, 9650 (35.3%) and 558 (34.5%), respectively, contained stigma statements, the highest proportions among age groups. Stigma statements were more common among people in peripheral cities versus other locations; this may suggest directions for educational activities. For example, young people’s limited knowledge of sexual health and lack of understanding of HIV/AIDS may lead to stigma, such as peril and fear [38]. The high number of tweets may indicate that this demographic could benefit from more educational activities. It should be noted that the people in their 20s whose tweets were recorded in 2011 were not the same people in their 20s whose tweets were recorded in 2023, and these different groups of people could have different relationships both to HIV and social media.

The label category made up a proportionately greater part of antistigma tweets than stigma tweets. This could represent a desire to avoid othering sexual minority groups. Some tweets condemned discrimination against people infected with COVID-19, citing past discrimination against people with HIV and lesbian, gay, bisexual, transgender, and queer people; such tweets linking labeling of people with HIV to people with COVID-19 could have contributed to the greater representation of the label category in antistigma versus stigma tweets.

Although the percentage and number of tweets from HCPs with stigma statements were small, they may have more impact than those from non-HCPs and should be considered important. It may be worthwhile to develop ways to amplify the influence of HCPs who transmit antistigma statements, for example. Organizations that promote awareness (including professional and academic institutions dedicated to infectious disease) could work together or in parallel with HCPs who wish to disseminate antistigma messages addressing all types of stigma. In addition, misinformation has spread, leading to HIV being conflated with COVID-19, including the false rumor that HIV was contained in COVID-19 vaccines [39]. Therefore, it is necessary to consider how knowledgeable recipients of information may be before disseminating research information.

### Comparison With Prior Work

The percentage of stigma messages in this study (n=574,687, 28.5%) was similar to that seen in English-speaking countries [18]. Brown et al [18] reported that label (34%) and insult (31%) were particularly high among stigma messages in English-language tweets, whereas peril (n=271,614, 47.3%) and fear (n=158,328, 27.6%) were high in this study, suggesting that the nature of HIV stigma may differ between the populations. Differences in stigma perceptions across nations have been found in previous research; in a global survey, concern about disclosing one’s HIV status was notably higher in the Asia-Pacific region versus North America [40]. In addition, since the incidence rate is lower in Japan than in other countries [41], HIV is perceived as a “foreign” disease [42], and the overall awareness of and familiarity with the disease are low; therefore, members of the general public may not be aware of any people with HIV in their lives, and this unfamiliarity can readily lead to fear and misunderstanding. Based on the results of the “Public Opinion Survey on HIV Infection and AIDS” [3], it is highly possible that although medical advances have made drugs more effective for treating the disease, the concept of “undetectable equals untransmittable” (U=U) is not yet sufficiently widespread among the general public. More detailed studies on the reasons for differences in stigma across countries are needed. Previous reports showed that understanding U=U improves the acceptance of people with HIV [43], so disseminating this concept and other accurate information via influencers may be a solution to reduce stigma among younger generations. Conducting regular awareness campaigns such as short movies illustrating the U=U concept on social networking sites may help, as such approaches were effective in reducing COVID-19–related stigma [44].

This study has several limitations. User bias is a potential limitation; although Japan has many X users [23], those users do not necessarily accurately represent the entirety of society. The user attributes recorded are based on self-declaration by the users and cannot be verified; both intentional misrepresentation and outdated information (eg, ages in profiles) may be present, and there is potential for bias between users who choose to self-disclose information and users who do not. Further, the selection of tweets in Japanese may not necessarily represent the attitudes of Japan, as tweets in any language may be posted from different places. There are cases where certain users tweet very frequently (bots, advertisements, etc), which may influence the statistical results, although we attempted to eliminate consideration of such accounts. An additional limitation includes potential bias in the sample of tweets. HIV-related tweets were limited to those published before 2024 (and not deleted at the time of data retrieval) with specific keywords, and potentially HIV-related tweets that did not mention specific keywords were not included in the study. In particular, antistigma may be tweeted in the form of replies, in which case, there is likely no mention of keywords, which may lead to the underestimation of antistigma tweets. Finally, there is the possibility of classification bias. Tweet classification was carried out through discussions within the team, but there were tweets with unclear intentions or tweets that were difficult to interpret, and decisions ultimately depended on the interpretation of individuals who annotated a certain label. Furthermore, the trained models and rule-based models are imperfect, with the AUC values reported (0.72 for the stigma model and 0.77 for the antistigma model) indicating moderate performance; there may be some misclassification, which may not be random and therefore biased toward a particular category. Future work can refine the training dataset by oversampling underrepresented categories or using transfer learning from models pretrained on larger datasets.

### Conclusions

This study contributes to a better understanding of the types and attributes of HIV stigma in Japan and illustrates the usefulness of social media for describing stigma. The results indicate that HIV stigma is still prevalent, particularly among young adults, who were not alive during the early days of the AIDS panic. This information can be useful in the future development of more effective educational activities. To respond more accurately to the Japanese public’s educational needs in the future, it may be important to clarify how better to reach these X users (ie, which influencers they follow and support and what topics they are interested in) and how to approach them to consider more efficient educational activities in the future.

## Abbreviations

AUC: area under the curve
BERT: Bidirectional Encoder Representation From Transformers
HCP: health care practitioner
OR: odds ratio
U=U: undetectable equals untransmittable

## Data Availability

The datasets generated or analyzed during this study are available from the corresponding author on reasonable request.
